# Architecting an enterprise financial management model: leveraging multi-head attention mechanism-transformer for user information transformation

**DOI:** 10.7717/peerj-cs.1928

**Published:** 2024-03-15

**Authors:** Wan Yu, Habib Hamam

**Affiliations:** 1 Huanghe Science and Technology University, Zhengzhou, Henan, China; 2 Faculty of Engineering, Uni de Moncton, Moncton, Canada; 3 International Institute of Technology and Management (IITG), Avenue des Grandes Ecoles, Libreville, Gabon; 4 Bridges for Academic Excellence, Tunis, Centre Ville, Tunisia; 5 Department of Electrical and Electronic Engineering Science, School of Electrical Engineering, University of Johannesburg, Johannesburg, South Africa

**Keywords:** Financial management, Transformer, Reinforcement learning, GAN

## Abstract

Financial management assumes a pivotal role as a fundamental information system contributing to enterprise development. Nonetheless, prevalent methodologies frequently encounter challenges in proficiently overseeing diverse information streams inherent to financial management. This study introduces an innovative paradigm for enterprise financial management centered on the transformation of user information signals. In its initial phases, the methodology augments the Transformer network and self-attention mechanism to extract features pertaining to both users and financial data, fostering a more cohesive integration of financial and user information. Subsequently, a reinforcement learning-based alignment method is implemented to reconcile disparities between financial and user information, thereby enhancing semantic alignment. Ultimately, a signal conversion technique employing generative adversarial networks is deployed to harness user information, elevating financial management efficacy and, consequently, optimizing overall financial operations. The empirical validation of this approach, achieving an impressive mAP score of 81.9%, not only outperforms existing methodologies but also underscores the tangible impact and enhanced execution prowess that this paradigm brings to financial management systems. As such, this work not only contributes to the state of the art but also holds promise for revolutionizing the landscape of enterprise financial management.

## Introduction

The great progress of development requires enterprises to have efficient financial management capabilities to adapt to quick business transaction processing. A good financial management system can help enterprises quickly meet the market demand and quickly deploy various resources throughout the enterprise (Sukenti, 2023). Therefore, the study of efficient and fast enterprise financial management systems has extremely high application value.

In addition, studying enterprise financial management helps assess and manage all kinds of risks faced by enterprises, including market risks, credit risks, liquidity risks, *etc*., to ensure steady enterprise development. Further analysis of financial data can provide decision support for enterprise leadership, help formulate strategic planning, optimize business models, and choose appropriate development directions  (Winarno, Agustina & Vinola, 2020). Studying financial management helps enterprises make wise investment and financing decisions, choose the most suitable financing methods and investment projects for enterprise development, and protect shareholders’ rights and interests to the greatest extent (Bustani, Khaddafi & Ilham, 2022). Studying financial management helps enterprises adapt to global competition, understand the characteristics of international markets, formulate global financial strategies, and expand international markets  (Klapper & Lusardi, 2020). Therefore, the study of enterprise financial management has extremely high theoretical value.

Enterprise financial management is a complex and multi-level research in which there are some difficulties and challenges (Mazur et al., 2021; Cui, 2023; Chen & Metawa, 2020). (1) The quality of financial data is uneven and diverse: Different enterprises may have large differences in the format, structure and specification of financial data, which leads to difficulties in data integration and analysis. (2) Finance is multi-layered: enterprise financial management involves multi-level decisions. Carrying out reasonable information transmission and decision coordination at different levels to support the realization of the enterprise’s overall financial goals is a complex issue. (3) Financial risk and uncertainty: Enterprises are faced with various and complex types of risks regarding market, credit, liquidity, *etc*. How to effectively quantify, identify and manage these risks and reduce uncertainty through financial management research is a challenging research direction (Abad-Segura et al., 2020; Aifuwa & Embele, 2019).

Around the above difficulties, many scholars have carried out a series of studies. Chen & Metawa (2020) posited that the swift advancement of IT has the potential to bolster organizational performance within the AIS and enhance the competitive edge of both enterprises and institutions. Nazah et al. (2022) organized and revamped the accounting table, enabling an efficient display of accounting codes and product-related data for information subjects using system queries. This enhancement aims to optimize budget management for enterprises. Ren (2022) asserted that American colleges had implemented comprehensive management and information systems encompassing budgeting, funding, analysis and decision-making. With the development of signal processing, signal conversion algorithms have become an important way to quantify financial information. Gao (2022) proposed an enterprise financial information system based on cloud technology, which helps enterprises build a powerful, simple operation and strong business expansion information system at low cost through cloud computing, deep learning and other technologies. Sitinjak et al. (2023) perfected the traditional financial information system by using the big data model based on the Meacher model.

In spite of the extensive research undertaken by the scholars above on financial information systems, persistent limitations and challenges remain. Primarily, current methodologies predominantly concentrate on singular factors in financial management, presenting challenges when dealing with multimodal information. Additionally, these approaches fall short of establishing swift connections and models between users and financial information, potentially impeding the flexibility and responsiveness of information systems. Furthermore, while innovative technologies have been introduced, further refinement is essential in certain areas, such as signal conversion algorithms and the utilization of big data models, to enhance the overall efficiency of financial information systems.

To address these issues, we propose an enterprise financial management method grounded in user information signal conversion. Recognizing that financial management involves diverse types of information, including multimodal data such as digital data, text, and images, we have employed a comprehensive approach to enhance both the Transformer network and the self-attention mechanism. The aim is to more efficiently extract features related to users and financial data, fostering a more comprehensive comprehension of various information types by the system. Subsequently, a reinforcement learning-based alignment method is introduced to swiftly reconcile disparities between financial and user information, enhancing semantic alignment. This method exhibits increased flexibility in adapting to different information modalities, ensuring coherence between the two sets of information. Lastly, a signal conversion method based on generative adversarial networks has been incorporated, allowing user information to play a more effective role in financial management. The objective of this phase is to enhance the efficiency of financial operations and elevate the decision-making process’s intelligence and accuracy by optimizing the utilization of user information.

Key contributions include:

1. Introducing a novel multimodal feature extraction method based on an enhanced Transformer to quantify both financial and user information.
2. Proposing a feature alignment method founded on reinforcement learning and a signal conversion method utilizing generative adversarial networks to model the interrelationships between users and finance.
3. Achieving superior performance when compared with other competitive methods on the Enterprise Finance Dataset.

## Related Works

### Signal processing techniques

Signal processing technology refers to a series of operations such as collecting, analyzing, processing, and extracting information, improving characteristics, recognizing patterns, and predicting the future behavior of signals. These signals can be sounds, images, texts, videos, or even complex biological signals, among others. The signal processing technology is widely used in communication, audio processing, image processing, biomedicals, finance, automatic control and other fields.

In the field of signal processing, the dLMS algorithm (Nassif et al., 2017; Nassif et al., 2018) usually has a slow convergence speed because the covariance matrix of the signal usually has a large eigenvalue spread. At the same time, dLMS proves that dLMS based on Newton’s method (dLMSN) has a faster convergence speed than the traditional dLMS algorithm, but its cost is the need to calculate the inverse matrix, which has high algorithm complexity. Furthermore, Hua et al. (2018) proposed the diffusion preconditioned LMS (dPLMS) based on preconditioning, which uses preconditioning operations to boost the convergence rate of the algorithm and approximately achieves the stable status of the dLMSN.

Financial management is signal processing research with careful consideration of multitasks. In the context of multitask parameter estimation, it is common to partition the nodes within the network into several clusters, with each cluster consisting of nodes assigned to perform a specific parameter estimation task. Chen, Richard & Sayed (2014) realized the collaborative estimation of multiple parameters by predicting the clustering information and the relationship between different tasks and fully considering the collaboration between clusters. Nassif et al. (2016) assume that the parameters estimated by nodes in different clusters are linearly correlated, and the linear constraint relationship between the parameters to be estimated is known in advance. Then, when the step size of the dLMS algorithm is small enough, each node can collaboratively achieve the optimal estimation of any accuracy. On the contrary, Chen, Richard & Sayed (2015) proposed that when there is no prior knowledge about different tasks in multitask scenarios, the unsupervised clustering strategy can adaptively adjust the combination strategy of each node according to the similarity of the estimation tasks of each node, so that each node can realize adaptive collaborative estimation.

### Current research status of financial management

Financial management technology refers to a series of methods and tools that apply information technology and related software tools to improve, optimize and automate the financial activities of enterprises. These technologies can help enterprises more efficiently carry out financial planning, financial analysis, budget control, cost management, risk assessment, investment decisions and other aspects of the work.

Berdiev (2023) believes that by using the information technology platform of the Internet, enterprises can carry out various commercial trade activities, make the form of trade more efficient, reduce the cost of commercial input, expand the scope of commercial activities, and continuously realize the diversification of commercial operations. Polzer, Nolte & Seiwald (2023) held the view that the swift evolution of the Internet has transformed work dynamics. Financial management is also undergoing continuous reform with the improvement of information technology. Yermack (2017) believed that in the “Internet +” market environment, the financial management structure of enterprises is also under continuous reform.

Gigli, Mariani & Trivellato (2018) proposed that in the process of transformation from a cash basis to an accrual basis, excessive bureaucratization of management, the complexity of organizational structure, limitations of IT system and regulatory loopholes will affect the reform and innovation of financial administration. Therefore, it is necessary to consider the establishment of a framework system from the aspects of system, organization and technology. Hoerlsberger (2019) put forward that the integration of IT has a great influence on universities, governments, enterprises, and management innovation not only through the construction of information but also through structural changes. To adapt to the development of digital trends, optimize and innovate in organizational structure, personnel setup and management mechanism, and establish a new management system  (Mohammad, 2018). Paterson & Wilson (2018) proposed an accounting system based on information technology, which simplifies daily tasks and work, simplifies financial reporting and disclosure, and changes the development of the whole financial management. Cooper, Holderness Jr & Sorensen (2019) proposed an automatic input, processing and output of information data based on intelligent robots to simplify repetitive work, liberate labour, and improve work efficiency.

While the methods mentioned above have introduced a range of innovations and enhancements, they also exhibit potential drawbacks and challenges. Certain financial management approaches still exhibit an excessive focus on a single factor, potentially overlooking the diversity of financial information and creating challenges in handling complex and multimodal scenarios. In addition, there is a failure in some methods to efficiently establish rapid connections and models between users and financial information, limiting the adaptability of information systems and leading to suboptimal responses to evolving user needs and changes. Addressing these shortcomings is imperative in future research and practical implementations to ensure that innovations in financial management systems are comprehensive and effective in meeting the evolving demands of enterprises and institutions.

The previously mentioned financial management methods, as highlighted in the literature, have undoubtedly brought about advancements in optimizing and automating financial activities. However, these approaches are not without their shortcomings, and addressing these limitations is crucial for the continued evolution of financial management systems. One common drawback observed in certain methods is the tendency to overly emphasize a singular factor, neglecting the diverse nature of financial information. This narrow focus may result in challenges when dealing with intricate and multimodal scenarios, potentially limiting the overall effectiveness of the financial management system. Furthermore, some existing methods exhibit a deficiency in establishing rapid connections and models between users and financial information. This limitation hampers the adaptability of information systems, leading to suboptimal responses to evolving user needs and dynamic changes in the financial landscape. The failure to efficiently integrate user interactions with financial data can impede the system’s ability to provide timely and relevant information, hindering its overall utility in practical applications.

The proposed method seeks to overcome these drawbacks by introducing novel approaches that address the identified shortcomings. By emphasizing a holistic perspective on financial information and adopting advanced modelling techniques, the proposed method aims to handle complex and multimodal scenarios more effectively. Additionally, it focuses on establishing efficient connections between users and financial information, enhancing the system’s adaptability to changing user needs. Through these innovations, the article contributes to a more comprehensive and effective financial management system that is better equipped to meet the evolving demands of enterprises and institutions in a dynamic market environment.

### Enterprise financial management method based on user information signal conversion

The existing financial management methods make it difficult to deal with the multimodal information in the system. They cannot realize the rapid connection and modelling between users and financial information. In this article, an enterprise financial management method based on user information signal conversion is proposed. Aiming to align user information and financial information, this method proposes a multimodal feature extraction method based on an improved transformer, a feature alignment method by reinforcement learning, and a signal conversion method by the generative adversarial network. Combined with the signal conversion of user information, the mutual relationship between users and finance is modelled.

### Multimodal feature extraction method based on improved transformer

**Figure 1:**
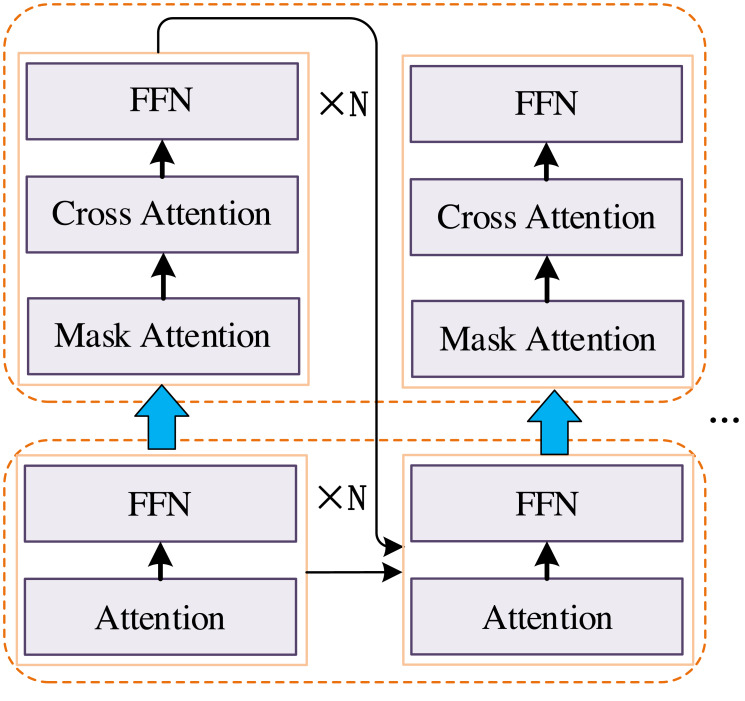
Structure of our improved transformer.

In this article, the Transformer-based multimodal feature extraction method is used as a feature quantification method for financial information and user information, and its codec structure has a strong receptive field, as shown in Fig. 1. Transformer is a kind of codec. By calculating the similarity between tokens, it extracts semantic information and realizes the feature language expression. The Transformer network improves the malleability of LSTM, which can effectively model the relationship between two elements with a large distance and take into account the relationship mining between short-term elements. In addition, the basic unit of the Transformer is matrix operation, which avoids the cyclic calculation of elements and saves computing resources during training and testing.

 Through enhancements to the network structure, our objective is to amplify the information exchange between the encoder and decoder, thereby narrowing the semantic gap induced by cross-modal transformations. Within the refined Transformer network, the multi-head attention mechanism (MHSA) assumes a pivotal role as a fundamental component. This mechanism endows the network with heightened semantic modelling capabilities. Our emphasis lies in fostering close collaboration between the encoder and decoder, facilitating more effective sharing of crucial information through direct exchange mechanisms. This strategic approach mitigates potential information loss or distortion during cross-modal transformations, enhancing the system’s adaptability when confronted with diverse data modalities. Particularly noteworthy is the framework of the multi-head attention mechanism, wherein the network gains greater flexibility to concentrate on varied positions and features. This flexibility ensures a more comprehensive capture of semantic information from input data, a critical aspect for cross-modal transformation tasks given the distinct semantic features associated with different modalities. The structural refinements undertaken are geared towards elevating the Transformer network’s performance in processing multimodal data, minimizing semantic gaps, and empowering the system to adeptly comprehend and transform diverse information types. This optimization yields a positive impact on bolstering the overall efficiency and applicability of the system. In principle, the self-attention mechanism uses the Query feature (Q) to weight the Key-Value feature group. This process involves computing a similarity matrix between the Query feature and the Key feature (K) and then using this similarity matrix to weight the Value feature (V). This is computed as follows:

(1) \begin{eqnarray*}Q& =x{W}^{Q}\end{eqnarray*}

(2) \begin{eqnarray*}K& =x{W}^{K}\end{eqnarray*}

(3) \begin{eqnarray*}V& =x{W}^{V}\end{eqnarray*}

(4) \begin{eqnarray*}\text{Attention}& =\text{softmax} \left( \frac{Q{K}^{T}}{\sqrt{{d}_{k}}} \right) V.\end{eqnarray*}

**Figure 2:**
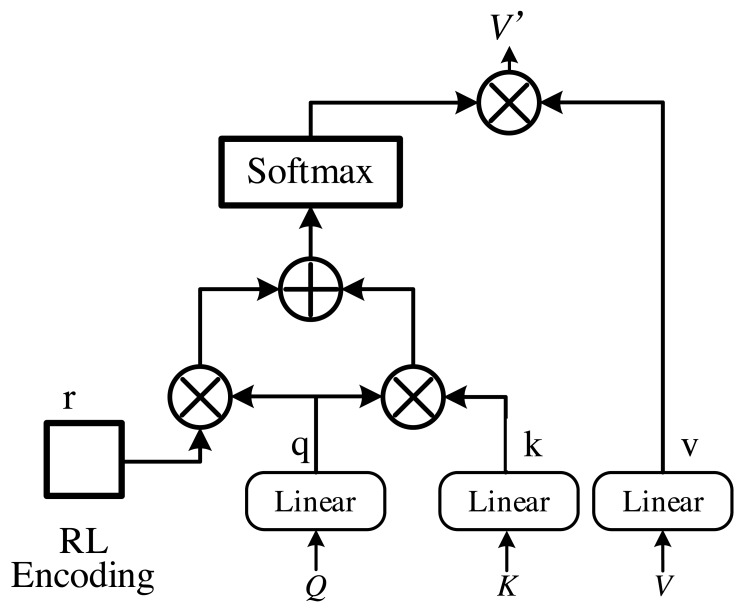
Improved self-attention.

*W*^*Q*^, *W*^*K*^ and *W*^*V*^ are the learnable parameters, *x* is the sequence feature vector, and *d*_*k*_ is the dimension of the *Q* feature and *K* feature. The above equation is used to compute the self-attention mechanism. Furthermore, in order to effectively leverage the sequencing of financial information and augment the capabilities of the self-attention mechanism, we introduce an enhanced self-attention mechanism, depicted in Fig. 2. Subsequently, the enhanced self-attention mechanism is employed to construct the MHSA, which is illustrated in the following equation:

(5) \begin{eqnarray*}& & \text{MultiHead} \left( Q,K,V \right) = \left[ {\text{head}}_{1},\ldots ,{\text{head}}_{n} \right] W\end{eqnarray*}

(6) \begin{eqnarray*}& & {\text{head}}_{i}=\text{Attention} \left( {Q}_{i},{K}_{i},{V}_{i} \right) \end{eqnarray*}

*Q* means query features, *K* refers to key features and *V* denotes value features. In addition, [.] means concatenate.

As we know, long short term memory (LSTM) is a competitive method. We apply the Transformer because it employs a self-attention mechanism, enabling simultaneous processing of all positions in the input sequence, a departure from LSTM’s sequential and step-by-step processing. This characteristic enhances the Transformer’s efficiency in handling lengthy sequences, as it facilitates more parallel computations. The self-attention mechanism equips Transformer with the ability to establish global long-range dependencies within input sequences. In contrast, LSTM may encounter challenges related to vanishing or exploding gradients, limiting its effectiveness in capturing extended sequence dependencies. The self-attention mechanism in Transformer enhances model interpretability, providing a transparent understanding of each input position’s contribution to the output. Conversely, LSTM’s internal state and gating mechanism may present relatively higher complexity for interpretation.

### Feature alignment method based on reinforcement learning

Through the Transformer-based multimodal feature extraction method, we can obtain the financial features of the enterprise and the user features of the enterprise. However, these two features belong to different semantic fields, which directly leads to the subsequent feature matching is not differentiable. Moreover, there is a large gap between the evaluation index of our method and the commonly used cross-entropy loss, which cannot directly improve the model through the training of the model, resulting in the results of training and testing being difficult to be consistent, and errors will continue to accumulate in the process of alignment.

Reinforcement learning (RL) is an artificial intelligence (AI) method that focuses on how an intelligent agent can learn to make decisions in specific scenarios through interaction with the environment to maximize the overall reward (or minimize the penalty). In reinforcement learning, an intelligent agent performs a sequence of actions and subsequently ADAPTS its approach based on the response (reward or punishment) of the environment to improve the outcome over a longer period. This learning process usually involves a trial-and-error process, where the agent gradually improves its behaviour by constantly trying various strategies.

**Figure 3:**
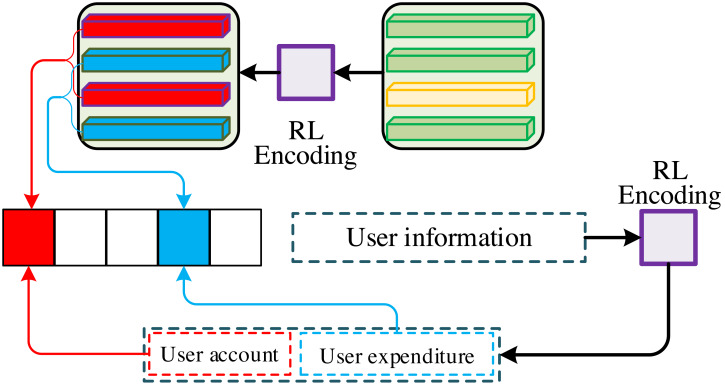
Signal conversion method based on reinforcement learning.

The core of the feature alignment by reinforcement learning is to regard signal conversion as a reinforcement process, as shown in Fig. 3, which causes the training purpose of the model to be changed to minimize the negative value of the expected reward. The formula is as follows:

(7) \begin{eqnarray*}L \left( \theta \right) =-{\mathrm{E}}_{w\sim {p}_{\theta }} \left[ r \left( {w}^{s} \right) \right] .\end{eqnarray*}

Since the evaluation indexes of signal conversion are not differentiable, the constrained gradient of *L* (*θ*) can be obtained based on Monte-Carlo theory, as shown in the following formula:

(8) \begin{eqnarray*}{\nabla }_{\theta }L \left( \theta \right) & =-{\mathrm{E}}_{w\sim {p}_{\theta }} \left[ r \left( {w}^{s} \right) {\nabla }_{\theta }\mathrm{log}{p}_{\theta } \left( {w}^{s} \right) \right] \end{eqnarray*}

(9) \begin{eqnarray*}{\nabla }_{\theta }L \left( \theta \right) & =-{\mathrm{E}}_{w\sim {p}_{\theta }} \left[ \left( r \left( {w}^{s} \right) -b \right) {\nabla }_{\theta }\mathrm{log}{p}_{\theta } \left( {w}^{s} \right) \right] .\end{eqnarray*}

In the training of financial information and personal information signal transformation, *b* is usually defined as the reward value predicted by the model at the current time, denoted as *r*(*w*′), and its formula is as follows:

(10) \begin{eqnarray*} \frac{\partial L \left( \theta \right) }{\partial {s}_{t}} = \left( r \left( {w}^{s} \right) -r \left( {w}^{{^{\prime}}} \right) \right) \left( {p}_{\theta } \left( {w}^{s}{|}{h}_{t} \right) -1 \right) .\end{eqnarray*}

Among them, the reward value is usually calculated using evaluation metrics to better maintain the consistency of model training and testing performance.

### Signal conversion method by GAN

To model the relationship between users and finance, we propose a signal conversion method by generative adversarial networks.

**Figure 4:**
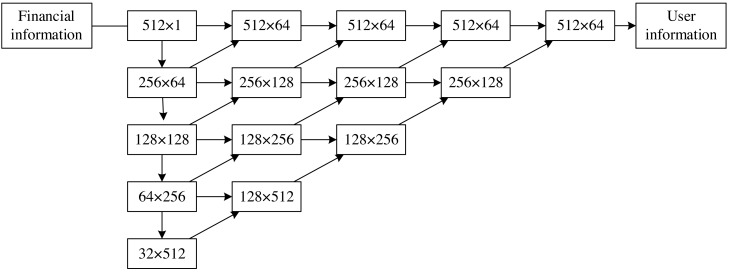
Structure of the generated network.

By learning the probability distribution of financial signal data, GAN can convert the signal input by the user into a financial signal so that it can be identified as a real financial signal through the discrimination network. The framework of the generated network is shown in Fig. 4, and a convolutional neural network (CNN) based on the Unet++ structure is employed. The structure is divided into three parts: encoding path, decoding path and skip connection. In the figure, the number in each rectangular box represents the length of the data multiplied by the number of channels. The encoding part down-samples the signal, and each step contains a convolution module and a max pooling layer to realize the extraction of signal features. The decoding part is opposite to the encoding part, with the difference that an upsampling layer replaces the max pooling layer. The utilization of upsampling layers serves to increase the spatial dimension of the feature map, facilitating the restoration of details and spatial resolution within the input image. Simultaneously, the decoding segment requires the integration of multi-scale features from the encoding section to effectively restore finer details. The upsampling layer functions as a mechanism for amalgamating these multi-scale features.

In contrast, the maximum pooling layer in the encoding part contributes to information loss. The incorporation of upsampling layers minimizes information loss to a significant extent, allowing for the optimal preservation of crucial features. The skip connection part is also different. It is different from the Unet structure that directly connects the feature map of the encoding path and the decoding path. Still, it integrates the convolution module into the skip connection and fuses the features of the next stage of convolution to optimize the feature fusion step. The channels of the feature need to be adjusted by the convolution; each convolution includes two one-dimensional convolution layers: the batch normalization (BN) layer and the ReLU activation layer. Finally, the four upsampled feature maps were fed into a one-dimensional convolutional layer to output data with the same dimension.

**Figure 5:**
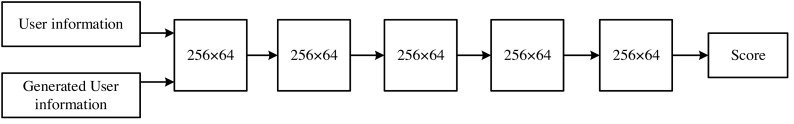
Discriminative network structure.

The discriminant network is used to estimate the probability that the user data converted by the generation network is consistent with the original financial data, which is essentially a binary classifier. The network is presented in Fig. 5. The number in each rectangular box in the figure represents the length × the number of channels of the data after the convolution module. The original financial signal and the generated user signal are concatenated and input into the discriminative network. The network first uses a one-dimensional convolution layer with convolution kernel size three and step size 1 to extract shallow features and then uses four identical modules. Each module includes a 1-D convolution layer with convolution kernel size four and step size 2, a BN and an activation layer. Finally, a one-dimensional convolutional layer is used to convert the number of channels to 1, and the sigmoid function is utilized to achieve the classification result. The LeakyReLU activation function is used to prevent overfitting, and the negative slope is set to 0.2 to ensure that the gradient transfer is simpler.

## Experiment and Analysis

### Dataset and implement details

We utilized the Enterprise Finance Dataset to assess the effectiveness of the enterprise financial management method based on user information signal conversion. This dataset was sourced from attachments within company financial reports submitted to the commission and is accessible on Zenodo. The dataset primarily includes financial statements, offering a more concise representation compared to the comprehensive financial statements and the Notes dataset. The latter comprises both numerical and narrative disclosures for all financial statements and accompanying notes. The information aligns consistently with the financial reports “filed” by each registrant. The data is organized in a clear format, facilitating users in the analysis and comparison of company disclosures over time and among registrants.

Moreover, the dataset incorporates supplementary fields, such as standardized industry classifications for companies, streamlining the utilization of data. Given that Transformers, reinforcement learning, and GANs are widely utilized for big data training, we intend to establish the environment and conduct model training using CPU: Xeon(R) E5-2640 v4 and GPU: 4*Nvidia Tesla V100. Tensorflow will serve as the deep learning framework for these endeavours. The specifics of the experimental parameters can be found in Table 1. We set the learning rate decay term of the model to 0.095 and the initial learning rate to $8\times 10\hat {}(-4)$, epoch set to 80, batch size set to 40, and SGD is used as the optimizer.

**Table 1:** Implementation parameters.

Parameters	Value
Initial learning rate	8 × 10^−4^
Epoch	80
Batch-size	40
Decay	0.95
Gradient descent method	SGD
Image input size	380 × 380
Image feature dimension	1024

Since the enterprise financial management method is a multimodal task, we adopt the mean square Error (mAP) and F-measure as the evaluation criteria of the method, which are calculated as follows:

(11) \begin{eqnarray*}& & {V}_{P}= \frac{gt\bigcap pr}{pr} \end{eqnarray*}

(12) \begin{eqnarray*}& & {V}_{R}= \frac{gt\bigcap pr}{gt} \end{eqnarray*}

(13) \begin{eqnarray*}& & F= \frac{2\times {V}_{P}\times {V}_{R}}{{V}_{P}+{V}_{R}} \end{eqnarray*}

(14) \begin{eqnarray*}& & mAP= \frac{1}{N} \times \sum {V}_{P}\times {V}_{R}.\end{eqnarray*}

In the equation, pr refers to the result of the method and gt denotes the true value present in the dataset. In addition, we evaluate the performance of the enterprise financial management by the amount of calculation, the number of model parameters, and the operation time.

### Compare our detection method with others

**Figure 6:**
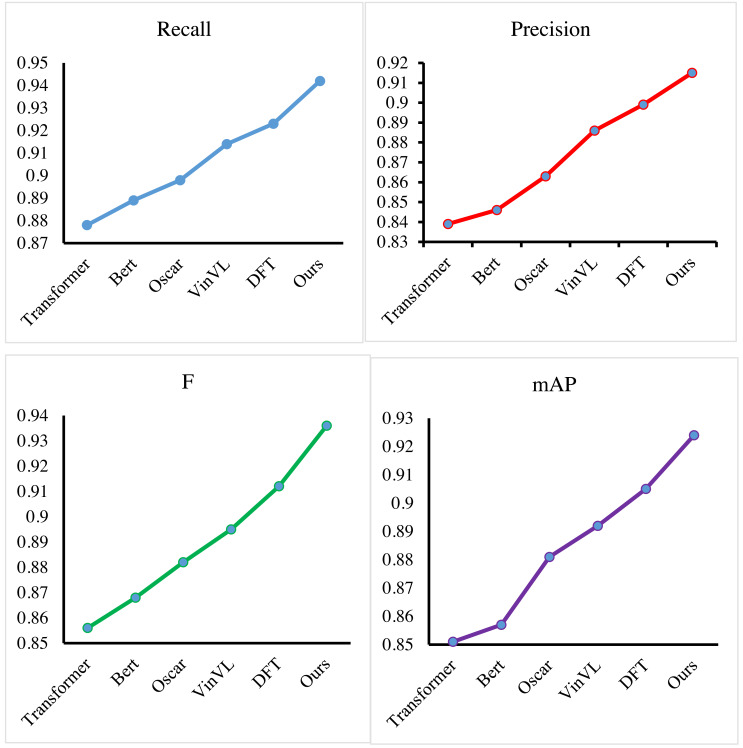
Compare our method with others.

First, we conduct experiments on the improved Transformer-based multimodal feature extraction method on the Enterprise Finance dataset. We select some excellent feature models, such as Transformer (Vaswani, Shazeer & Parmar, 2017), Bert (Deepa, 2021), Oscar (Li, Yin & Li, 2020) and, VinVL (Zhang, Li & Hu, 2021) and DFT (Zhang, Xie & Ding, 2023), and compare the performance. The results are presented in Fig. 6 and Table 2. We can conclude that our method obtains the highest value in all evaluation metrics, which is 0.942 for recall, 0.915 for precision, 0.936 for F-measure, and 0.924 for mAP, while comparing with other algorithms. Compared with the Transformer, our method improves the mAP value of the model by more than 7%, mainly because we improve the Transformer and optimize the self-attention mechanism. Compared with BERT, our method has a lead of more than 6%. BERT is almost the same as a Transformer in principle, so the performance is comparable between the two. Oscar and VinVL are models that are pre-trained with big data and have better adaptability to multimodal tasks, and our method still obtains more than a 3% improvement in the mAP score. Compared with the latest DFT method, which can obtain more than 90% of the mAP value by excellent model performance, our method still obtains about a 2% advantage. For the extraction of enterprise financial features and user features, the multimodal feature extraction by the improved Transformer proposed in this article has great advantages. Through the improved self-attention mechanism and recurrent Transformer structure, the financial features and user features can be effectively extracted and aligned.

**Table 2:** Compare our detection method with other methods.

Methods	Recall	Precision	*F*	mAP
Transformer	0.878	0.839	0.856	0.851
Bert	0.889	0.846	0.868	0.857
Oscar	0.898	0.863	0.882	0.881
VinVL	0.914	0.886	0.895	0.892
DFT	0.923	0.899	0.912	0.905
Ours	0.942	0.915	0.936	0.924

**Figure 7:**
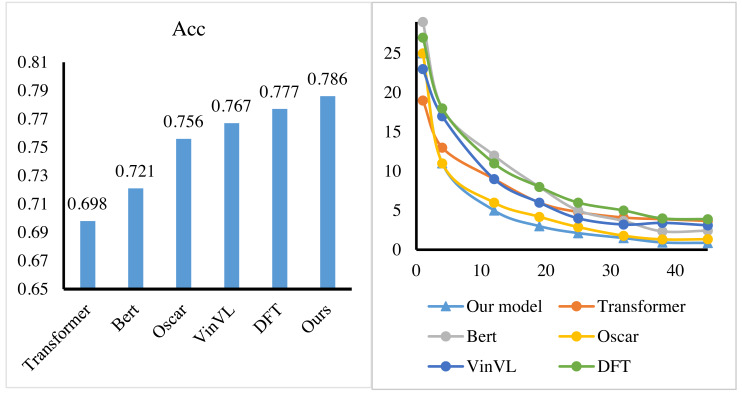
The performance of our signal process method.

Then, we implemented the performance test of the reinforcement learning-based feature alignment method on the dataset. The alignment yield mainly evaluates feature alignment. Therefore, the conversion rate of feature alignment was used as the index in this experiment. We still compare our method to Transformer (Vaswani, Shazeer & Parmar, 2017), Bert (Deepa, 2021), Oscar (Li, Yin & Li, 2020), VinVL (Zhang, Li & Hu, 2021) and DFT (Zhang, Xie & Ding, 2023). Figure 7 shows that the feature alignment method by RL achieves the highest feature alignment yield, that is, 78.6%, which reaches the most advanced level in the world. In addition, the training process is recorded, and the convergence graph is generated. In Fig. 7, the training process of the proposed method is very smooth, and the lowest loss value can be obtained, which fully demonstrates the stability and scalability of the proposed method to data.

**Figure 8:**
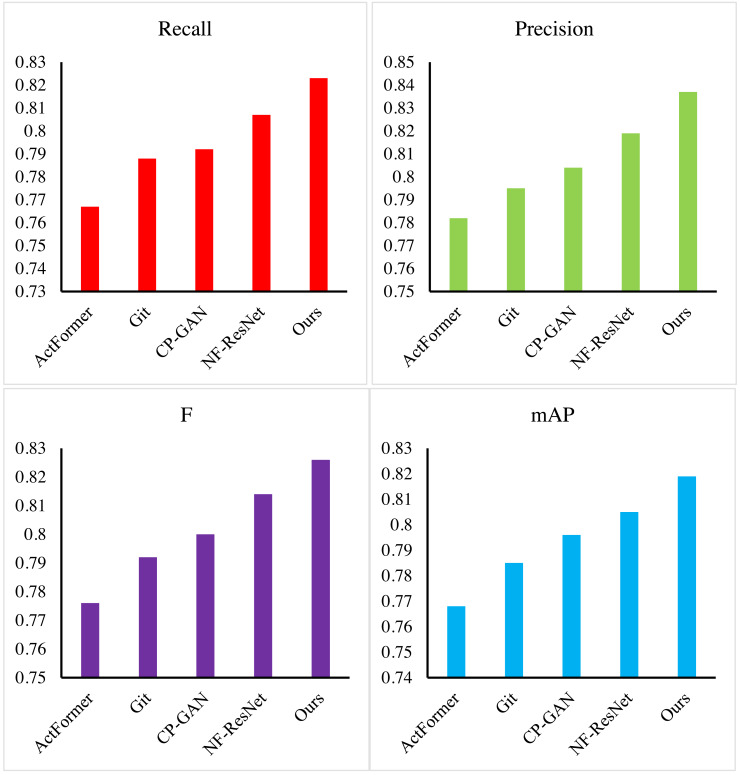
The results of our method.

After validating the multimodal feature extraction method based on the improved Transformer and the signal conversion method grounded in reinforcement learning, we further assessed our proposed signal conversion method using generative adversarial networks (GAN) on the dataset. Our method was benchmarked against notable models, including ActFormer (Xu, Song & Wang, 2023), Git (Wang, Yang & Hu, 2022), CP-GAN (Skariah & Thomas, 2023), and NF-ResNet (Khan et al., 2022). Evaluation metrics encompassed recall, precision, F-measure, and mAP, with results presented in Fig. 8 and Table 3.

**Table 3:** Compare our detection method with other methods.

Methods	Recall	Precision	*F*	mAP
ActFormer	0.767	0.782	0.776	0.768
Git	0.788	0.795	0.792	0.785
CP-GAN	0.792	0.804	0.800	0.796
NF-ResNet	0.807	0.819	0.814	0.805
Ours	0.823	0.837	0.826	0.819

 Our method demonstrated exceptional performance, achieving the highest values of 0.823 in recall, 0.837 in precision, and 0.826 in F-measure across all evaluation metrics. In comparison with ActFormer, our method showcased a more than 5% improvement in mAP score and a 5% boost in the F-measure. Against Git, our method exhibited over a 3% F-measure lead and a 3.4% mAP value increase. In contrast to CP-GAN, our method outperformed in all aspects, with all evaluation indexes surpassing 2%. Finally, when compared with NF-ResNet, our method enhanced the mAP score by 1.4% and the F measure score by 1.2%. Leveraging a GAN model based on Unet with fewer layers and feature processing steps, our proposed signal transformation method demonstrated notably superior performance compared to other methods.

**Figure 9:**
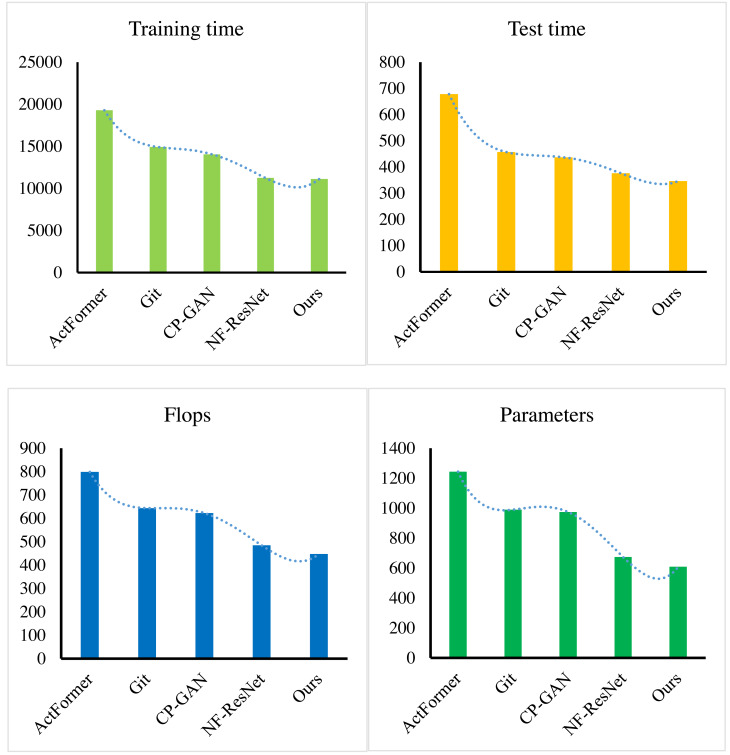
Model efficiency comparison with other methods.

The amalgamation of the three methods above culminated in the successful implementation of an end-to-end enterprise financial management system. The system evaluation metrics encompassed model parameter quantity, inference time, Flops (floating-point operations), and training time. As depicted in Fig. 9, the approach proposed in this article exhibited optimal performance, particularly in terms of training and testing time, as well as model parameter quantity. Our method not only streamlined the model by reducing the number of parameters but also significantly shortened inference and training times, ultimately enhancing the system’s efficiency. This results in expedited decision-making and heightened responsiveness in practical applications, providing enterprises with more efficient financial management services.

Moreover, the reduction in Flops and model parameters enhances the computational efficiency of the system, opening up adaptable deployment options for resource-constrained environments. The comprehensive approach presented in this article demonstrates superior performance across various dimensions, offering practical solutions for the development and application of enterprise financial management systems. Figure 9 visually underscores the excellence of our method across diverse evaluation metrics, serving as a valuable reference for future research and development endeavours in the realm of financial management systems.

## Discussion

In this article, we introduce a novel enterprise financial management approach centred around user information signal conversion. The primary goal is to address the challenge of handling multimodal information in financial management. We progressively enhance the stages of feature extraction, feature alignment, and signal conversion by incorporating an improved Transformer-based multimodal feature extraction method, a reinforcement learning-based feature alignment method, and a generative adversarial network-based signal conversion method. These methodological refinements aim to bridge the semantic gaps between financial information and user information, establish a reciprocal relationship between the two, and ultimately optimize financial management systems.

The experimental findings underscore the effectiveness of our proposed method, yielding an mAP score of 81.9%. This outcome substantiates the significant impact of our multi-faceted and comprehensive approach to managing multimodal information and refining financial management processes. This innovative method holds promise in furnishing enterprises with more streamlined and intelligent financial management solutions, fostering adaptability in the ever-evolving business landscape.

Despite the enhancements made to enterprise financial management methods, there exist notable shortcomings that require urgent attention and improvement. The substantial demand for computing resources in our proposed approach poses limitations on its feasibility in certain environments. The heightened complexity of the Transformer network and self-attention mechanism necessitates more advanced skills for effective management and maintenance.

Moving forward, we will persist in refining and expanding this approach to better cater to the evolving demands of enterprises in the realm of financial management.

While the introduced enterprise financial management approach, centred around user information signal conversion and leveraging advanced techniques, demonstrates promising results in optimizing financial systems, its practical implementation faces notable challenges. The substantial demand for computing resources poses feasibility issues in resource-constrained environments, necessitating a thorough exploration of resource-efficient alternatives. The heightened complexity of the Transformer network and self-attention mechanism highlights the importance of addressing potential skill gaps among personnel. Additionally, integration challenges with existing financial management systems, scalability concerns, regulatory compliance, and user acceptance are vital aspects that require careful consideration. A comprehensive cost-benefit analysis and a clear roadmap for future refinements will be crucial in enhancing the method’s overall viability and addressing the identified shortcomings for broader industry adoption.

## Conclusion

To cater to the dynamic requirements of sophisticated enterprise information systems, this article proposes an innovative paradigm for enterprise financial management. At the core of this approach lies the transformation of user information signals, effectively addressing the challenge of managing diverse modalities of information within the financial domain. Through enhancements such as multimodal feature extraction utilizing an optimized Transformer, a reinforcement learning-based feature alignment method, and a signal conversion method employing generative adversarial networks, we sequentially refine feature extraction, feature alignment, and signal conversion processes. These enhancements play a pivotal role in bridging the semantic gap between financial information and user data, enabling comprehensive modelling of their interrelation. Ultimately, these advancements pave the way for the optimization of the financial management system. Experimental results underscore the effectiveness of our method, attaining an mAP score of 81.9%, highlighting its potential to significantly elevate the performance of enterprise financial management systems. In our future endeavours, we aspire to delve into advanced research and implement cutting-edge algorithms and technologies to reduce dependence on hardware resources. This strategic approach aims to enhance the scalability and adaptability of our methods. Through meticulous refinement of feature extraction, alignment, and signal conversion steps, our primary objective is to elevate the overall performance of the system.

##  Supplemental Information

10.7717/peerj-cs.1928/supp-1 Supplemental Information 1 Code

10.7717/peerj-cs.1928/supp-2 Supplemental Information 2 DataSet for the paper

## Additional Information and Declarations

### Data Availability

The following information was supplied regarding data availability: The data is available at Zenodo: Tom. (2023). Enterprise Financial Dataset [Data set]. Zenodo. https://doi.org/10.5281/zenodo.10013148. The code is available in the Supplementary File.
